# Lower mortality rate in health workers and their families infected with COVID-19 associated pneumonia in Quetta, Baluchistan

**DOI:** 10.12669/pjms.37.7.4060

**Published:** 2021

**Authors:** Momina Aslam, Muhammad Tahir Zehri, HabibUllah Mandoklel, Rehana Kamal, Mohammad Din

**Affiliations:** 1 Momina Aslam, M.Phil. Department of Biotechnology, Sardar Bahadur Khan Women University, Quetta, Baluchistan, Pakistan; 2 Dr. Muhammad Tahir Zehri, FCPS. Department of Paeds Bolan Medical College, Quetta, Baluchistan, Pakistan; 3 Dr. Habib Ullah Mandokhel, FCPS. Department of E.N.T. Bolan Medical College, Quetta, Baluchistan, Pakistan; 4 Dr. Rehana Kamal, FCPS. Department of Gynaecology Bolan Medical College, Quetta, Baluchistan, Pakistan; 5 Dr. Mohammad Din, PhD. (Microbiology), Department of Pathology, Bolan Medical College, Quetta, Baluchistan, Pakistan

**Keywords:** COVID-19 Pneumonia, SARS-CoV, MERS-CoV, Health workers, Mortality, ARDS

## Abstract

**Objectives:**

A bunch of pneumonia cases in Wuhan, China, was caused by a novel beta-corona virus, (COVID-19) in December 2019 from where it spread rapidly across the globe. The aim of the study was to find out relevant reasons and offered suggestions to reduce the risk of infection and check clinical outcomes of the infected healthcare workers.

**Methods:**

This study was conducted in COVID-19 Real Time Polymerase Chain Reaction (RT-PCR) laboratory, Bolan Medical Complex Hospital Quetta, Baluchistan from May to June 2020. Eight hundred (n=800) health workers and their families were included in this study. Data were obtained with standardized data collection forms shared by the WHO. Nasopharyngeal samples were collected following the WHO protocols. RNA was extracted and amplified using real time reverse transcriptase polymerase chain reaction (RT-PCR). Serum ferritin level, C-reactive protein, D-dimer and radiological results of the RT-PCR confirmed individuals were also recorded and analyzed.

**Results:**

Among (n=800) health workers and their families 457 (57.1%) were COVID-19 PCR negative, 332 (41.5%) positive and 11 (1.4%) individuals were PCR positive but asymptomatic carriers, 234 (29.5%) were male and 103 (12.9%) were female. Mortality rate in our study was very low, only three patients (0.87%) died of this COVID-19 pneumonia.

**Conclusion:**

Our results showed increased rate of positive cases with fortunately lower mortality rate, although this novel pneumonia was associated with acute respiratory distress syndrome ARDS and intensive care unit (ICU) admission. Timely decisions, risk awareness knowledge and supply of necessary equipments are inevitable for the control of such epidemics.

## INTRODUCTION

Corona viruses belong to the family Corona viridae and the order Nidovirales and mostly dispersed in humans and other mammals. These are enveloped, non-segmented and positive sense RNA viruses.^1^ The World Health Organization (WHO) has declared a novel corona virus ‘‘2019-nCoV’’ responsible for the current outbreak of pneumonia in Wuhan City, Hubei Province, China in December, 2019.^2^ In majority of the COVID-19 cases fever, cough and other clinical symptoms were followed by respiratory failure, cytokine storm and ultimately death.^3^Abnormal pathological findings which characterize these clinical findings were elevated liver function tests, leukopenia and increased ferritin level.^4^ Excessive mortality was associated with mitochondrial dysfunction.^5^

Thrombotic microangiopathy in the capillaries of the lungs was another factor that contributed to death.^6^

A novel corona virus disease–nCoV-2019 is a new corona virus able to spread via respiratory secretions particularly through sneezing and coughing, from one to another person and surface contact. Incubation period of the virus ranges from 1 to 14 days. Fever, which occurred in 99% of the infected individuals, fatigue, shortness of breath, bilateral patchy infiltration on imaging and dry cough were common, diarrhea, vomiting and nasal congestion were rare symptoms of the disease.^7^ Mortality rate of COVID-19 was 2% which is lesser to MERS and SARS.^8^ Health workers are always prone to infectious diseases. After knowing that COVID-19 may spread via asymptomatic carriers, the risk is increased.^7^ In a previous report the spreading rate of the disease to health workers was 29%.^9^ In Wuhan City, a surgery patient infected 14 health professionals even before fever onset.^10^

To the best of our knowledge, fewer studies are available yet to check COVID-19 related knowledge, clinical, laboratory, radiological characteristics and clinical outcomes of the infected healthcare workers in Quetta, Baluchistan. Therefore, this study was designed to fulfill this gap and hopefully it will spread more information regarding COVID-19 infection and its clinical features in the community.

## METHODS

Study population Eight hundred (n=800) health workers and their families were included in this study after review and approval by the Institutional Review Board (IRB) and institutional ethical committee (No. Estt-DA-11 BMCH-AP-1472-4, dated 24-04-2020. Sample size was calculated and interpreted in accordance with the “Principles and Procedures of Statistics: A Biometrical Approach”. An average family size ranged from 8-12 members. So mean was taken as 10 members per family. Expected population was 8,000 and its 10% = 800.^11^ Samples were collected from those patients who presented with pneumonia symptoms or had contact history with COVID-19 positive patients and were suspicious.

Present study was conducted in COVID-19 (RT-PCR) laboratory, Bolan Medical Complex Hospital Quetta, Baluchistan from May to June 2020. Nasopharyngeal samples were collected in 03 ml viral transport medium VTM (COPAN) USA, following the WHO protocols in complete personal protective equipment (PPE). Samples were labeled and transported to the PCR laboratory immediately in cold chain and processed within one hour of collection. Blood was collected in commercially available 5 ml Becton Dickinson BD Vacutainer Gel Tubes Clot Activator.

The data were obtained with standardized data collection forms shared by the WHO. Medical records and the laboratory data were then submitted in prepared datasheets. The patients’ detailed information, including age, gender, clinical signs and symptoms were recorded.

RNA was extracted using MGI nucleic acid extraction kit cat. No.1000021043 Wuhan MGI Tech Co, Ltd. China, according to the manufacturer’s instructions. Nucleic acid was amplified for open reading frame (ORF) gene and nucleocapsid (N) gene from the isolates using real time fluorescent RT-PCR BGI Cat. No MFG030010, China and SYSTAAQ Lot. No.73027, Belgium kits following the manufacturer’s protocols and the WHO guidelines.^12^ BIO-RAD C-1000 Touch thermo cycler Model No. CFX96 Optic Module Singapore was used in the study.

Serum ferritin level, C-reactive protein and D-dimer were recorded using immunofluorescence quantitative analyzer, Getein1100, Biotech. Inc. China and Mindray-CL-900i Chemiluminescence Immunoassay System, China. Data collected were analyzed using SPSS version 16.0 (IBM, Armonk, NY, USA). One way analysis of variance (one way ANOVA), unpaired T-test, chi-sqaure and DMR test for significance were applied.

### Ethical considerations

Present study was approved from the Institutional Bioethical Committee Bolan Medical Complex Hospital, Quetta, Baluchistan. All patients completed the questionnaires with informed consent.

## RESULTS

All the enrolled 800 patients responded to the questionnaires positively. In eight hundred (n=800) health workers and their families, 539 (67.4%) were male and 261 (32.6%) were female. Majority of the patients, 427(53.4%) were belonging to age group-2. Among these, 457 (57.1%) were COVID-19 PCR negative, 332 (41.5%) were COVID-19 PCR positive, 11 (1.4%) individuals were PCR positive but asymptomatic carriers (Table-I).

**Table I:** Frequency and percentage of PCR results in comparison to age groups and sex.

	*Frequency*	*Percentage*
Age groups		
1-20 Years	104	13.0
21-40 Years	427	53.4
41-60> Years	269	33.6
Sex		
Male	539	67.4
Female	261	32.6
Result		
Negative	457	57.1
Symptomatic positive	332	41.5
Asymptomatic positive	11	1.4

The most common clinical feature at the onset of illness was fever in 288 (36%) patients followed by cough 256 (32%). Other common clinical manifestations included, sore throat 77 (9.6%), body ache 158 (19.8%) and difficulty in breathing 101 (12.6%) (Table-II). Less common symptoms were sputum production, headache and diarrhea.

**Table II:** General signs and symptoms of the patients.

	*Frequency*	*Percentage*
Fever		
No	512	64.0
Yes	288	36.0
Cough		
No	544	68.0
Yes	256	32.0
Sore throat		
No	723	90.4
Yes	77	9.6
Body aches		
No	642	80.2
Yes	158	19.8
Difficulty in breathing		
No	699	87.4
Yes	101	12.6
X-ray		
Normal	480	60.0
Infiltrate	320	40.0

The results of C-reactive protein, S-ferritin and D-dimer of the tested health workers and their family members showed significant difference (P<0.05) among positive and negative tested results, Highest C-reactive protein, S-ferritin and D-dimer (263.75±7.32; 471.41±5.01 and 1.5230±0.4) was revealed in symptomatic positive health workers. Whereas, no significant difference (P>0.05) between negative and carrier (asymptomatic positive) health workers was observed (Table-III). No significant difference (P>0.05) was observed among age groups in the positive subjects. Highest C-reactive protein (273.34±12.97) was noted in above 40 age group and D-dimer (1.70±0.62) was noted in age group of 21-40 years (Table-IV). C-reactive protein and D-dimer showed no significant difference (P>0.05) between male and female positive health workers. Whereas serum ferritin was noted higher in males (P<0.05) (Table-V).

**Table III:** Levels of CRP, S-ferritin D-dimer in negative, symptomatic positive and asymptomatic positive (carrier) health workers.

*Results*	*Frequency (%)*	*CRP (mg/L)*	*S-ferritin (ng/ml)*	*D-dimer (ng/ml)*
Negative	457 (57.1)	8.5651±1.92b	180.18±3.40b	0.3721±0.091b
Symptomatic positive	332 (41.5)	263.75±7.32a	471.41±5.01a	1.5230±0.4a
Asymptomatic positive	11 (1.4)	7.5391±0.49b	191.09±2.32b	0.3573±0.01b
Total	800 (100)	114.45±5.49	301.19±5.82	0.8495±0.17

*ab different superscripts in the same column are significantly different (P<0.05).

**Table IV:** CRP, S-ferritin and D-dimer in different age groups.

*Age Group*	*N*	*CRP (mg/L)*	*S-ferritin (ng/ml)*	*D-dimer (ng/ml)*
Age 1-20 Years	36	215.43±22.54	442.50±19.55	0.78±0.04
21-40 Years	185	251.60±9.95	468.92±7.14	1.70±0.62
41-60> Years	122	273.34±12.97	458.43±9.60	1.36±0.54
Total	343	255.54±7.49	462.42±5.58	1.48±0.38

Analysis of serum variables in male and female patients.

**Table V:** Comparison of the tested serum parameters in male and female patients.

	CRP (mg/L)		S-Ferritin (ng/ml)		D-dimer (ng/ml)	

*Sex*	*Male*	*Female*	*Male*	*Female*	*Male*	*Female*
N	239	104	239	104	239	104
Mean	259.745±8.99	245.86±13.60	478.89±6.87	424.54±8.38	1.50±0.48	1.43±0.63
Significance	0.85 (N.S)		0.026*		0.9069 (N.S)	

**Fig.1:**
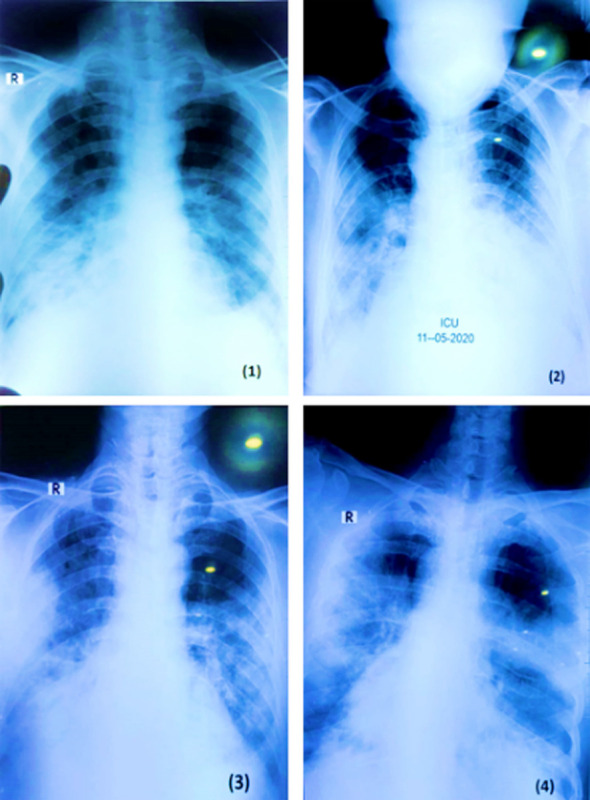
Chest radiographs of a 70 years old patient admitted in ICU.

Diffuse and bilateral infiltration was detected in the lungs of 320 (40%) symptomatic patients Table-II. Chest radiography of a 70 years old cardiologist admitted in ICU with three days interval from the onset of the disease, showing non homogenous shadowing in bilateral mid and lower zones, more marked in sub pleural region as are the indications of atypical pneumonia. Patient recovered from this novel beta-corona virus, (2019-nCoV) infection successfully (Fig.1).

## DISCUSSION

A large number of patients infected with (2019-nCoV) were identified in December 2019 in Wuhan, China from where it spread globally.^13^,^14^ Trade and tourism resulted in easier and faster spread of 2019-nCoV. Baluchistan (Quetta) was mainly affected from the influx of people from Iran because, it is located at the crossroads. Quetta is also among those cities where thousands of patients were screened for the disease. In the present study we screened eight hundred health workers and their family members who were on front line against this disease.

In some previous observations the infection rate of (2019-nCoV) pneumonia in health workers was from 15% to 18% and in some cases up to 20%^15^, in contrast to this study infection rate in our study was more than two-fold. Among 800 screened health workers and their families, 457 (57.1%) were COVID-19 PCR negative, 332 (41.5%) were COVID-19 PCR positive, 11 (1.4%) individuals were PCR positive but asymptomatic carriers. So, the total percentage of the positive individuals was 42.9%, which was very high.

The mortality rate of novel beta-corona virus, (2019-nCoV) has been significantly lower than SARS and MERS. The WHO reported the mortality rates of SARS -CoV about 9.6% and MERS-CoV 35.6% to 36%, while 3% for COVID-19.^16^-^18^ Clinically COVID-19 significantly resemble SARS-CoV and patients with severe disease developed acute respiratory distress syndrome (ARDS), went to ICU admission and oxygen therapy, since there was a short period between ARDS and hospital admission therefore, mortality rate was very high for 2019-nCoV, because 6 (15%) of 41 patients died.^1^ In contrast to these studies mortality rate in our study was very low, only three patients (0.87%) went to ICU and oxygen therapy and unfortunately died of this atypical pneumonia. This may be due to the genetic variability which occurs due to molecular changes like, point mutations (genetic drift) and RNA recombination (genetic shift) in the viral genome, 19 or because of the gradually developed immunity as, microbial invasion trigger the immune system which deliver an early response.^20^

A quick in vivo viral replication leads to uncontrolled inflammatory response in patients infected with COVID-19. Several studies have shown significant correlation between disease succession and the level of inflammatory response in COVID-19.^21^ In a previous study laboratory and immunological characteristics of 21 COVID-19 positive patients were analyzed, among these 12 patients showed serum ferritin level >800 µg/L (normal range: 30-400 µg/L). Moreover, blood was analyzed for other markers as well like highly sensitive C-reactive protein (hsCRP, median: 6.3-fold higher; p=0.003), elevated inflammatory biomarkers were directly proportional to the severity of the COVID-19 disease.^22^ In another study among the three groups of patients positive for COVID-19 showed significant differences in ferritin levels, especially between the mild and extremely severe groups. Furthermore, some other biomarkers such as D-dimer and LDH were also raised in severe and extremely severe cases.^23^

In parallel to the above-mentioned studies, our study showed significant difference (P<0.05) in C-reactive protein, S-ferritin and D-dimer among COVID-19 positive and negative patients. Highest C-reactive protein (263.75±7.32), S-ferritin (471.41±5.01) and D-dimer (1.5230±0.4) were revealed in symptomatic positive health workers. Whereas, no significant difference (P>0.05) was observed between negative and carrier (asymptomatic positive) individuals. Furthermore, no significant difference (P>0.05) was seen among age groups in the positive subjects. Highest C-reactive protein (273.34±12.97) was noted in above 40 age group and D-dimer (1.70±0.62) was noted in age group of 21-40 years. Similarly, sex in case of C-reactive protein and D-dimer also showed no significant difference (P>0.05) between male and female positive health workers. Whereas serum ferritin was noted higher in males (P<0.05).

Infected patients examined on X-rays and chest CT imaging showed unilateral or bilateral involvement which was complementary with viral pneumonia, and bilateral multiple lobular and sub- segmental consolidation areas were found in patients hospitalized in the ICU.^24^,^25^ In agreement to the above study the same observations were recorded in our study in about 40% of the symptomatic patients.

Health care workers, who are always on front line against outbreaks have all the time been at risk of the contagious diseases, and the spread of the COVID-19 has increased the risk many times,^8^ therefore health workers were especially focused in the present study to converge the attention of health policy makers towards the nature of the outbreaks, preventive measures and understanding their risk assessment at the right time. The most alarming problem we came across during this study was lack of pre-planned measures to evaluate the level of allied knowledge and anxiety among health care workers because, majority of the health workers were reluctant to participate in this war in the beginning. So, the health policy makers should concentrate on better risk communication and education for the control of epidemics especially for valuable health workers who are front line soldiers in health care system.

### Limitations of the study

Although it was a single setting study but healthcare workers were included from all health settings of the city and even from the peripheries of the province. Thus, the results cannot be generalized to the health workers of the other provinces. As more studies from other regions of the country are important to investigate risk awareness and mortality of the disease in the precious health workers on national level. However, this study will help policy makers and administrative authorities to take appropriate decisions for health workers.

## CONCLUSION

The outcome of this study showed increased ratio of positive cases with fortunately lower mortality rate in health care workers. Lower mortality rate seen in infected healthcare workers might be due to their frequent exposure to the disease and over-triggered immune system. But precautionary measures and risk perception must not be underestimated. It is important for health policy planners and decision makers to make timely decisions regarding self-protective behaviors, risk assessment knowledge and supply of essential equipments to the hospitals for the control of epidemics.

### Authors’ Contribution:

**MA & MD:** Conceived the presented idea, planning of the work and designed the basis of the manuscript.

**MD:** Responsible and accountable for the accuracy or integrity of the work.

**AH & HM:** Analyzed the data, and developed the theoretical part.

**MD & MA:** Contributed in PCR and ELISA lab Work.

**RK & MD:** Wrote the manuscript and provided data.

All authors contributed equally to this work and they provided critical feedback, helped shape the research and approved the final version of the manuscript for publication.

## References

[1] Huang C, Wang Y, Li X, Ren L, Zhao J, Hu Y (2020). Clinical features of patients infected with 2019 novel coronavirus in Wuhan, China. Lancet.

[2] Shirato K, Nao N, Katano H, Takayama I, Saito S, Kato F (2020). Development of genetic diagnostic methods for novel coronavirus 2019 (nCoV-2019) in Japan. Japanese J Infect Dis.

[3] Baden LR, Rubin EJ (2020). Covid-19 –The search for effective therapy. Editorial. N Engl J Med.

[4] Rosario C, Zandman-Goddard G, Meyron-Holtz EG, D'Cruz DP, Shoenfeld Y (2013). The Hyperferritinemic syndrome:macrophage activation syndrome, Still's disease, septic shock and catastrophic antiphospholipid syndrome. BMC Med.

[5] Shenoy S (2020). Coronavirus (Covid-19) sepsis:revisiting mitochondrial dysfunction in pathogenesis, aging, inflammation, and mortality. J Inflamm Res.

[6] Fox SE, Akmatbekov A, Harbert JL, Li G, Brown JQ, Vander Heide RS (2020). Pulmonary and cardiac pathology in African American patients with COVID-19:an autopsy series from New Orleans. Lancet Respir Med.

[7] Lai CC, Shih TP, Ko WC, Tang HJ, Hsueh PR (2020). Severe acute respiratory syndrome coronavirus 2 (SARS-CoV-2) and corona virus disease-2019 (COVID-19):The epidemic and the challenges. Int J Antimicrob Agents.

[8] Taghrir MH, Borazjani R, Shiraly R (2020). COVID-19 and Iranian Medical Students;A Survey on Their Related-Knowledge, Preventive Behaviors and Risk Perception. Arch Iran Med.

[9] Wang D, Hu B, Hu C, Zhu F, Liu X, Zhang J (2020). Clinical characteristics of 138 hospitalized patients with 2019 novel coronavirus–infected pneumonia in Wuhan, China. JAMA.

[10] Chang D, Xu H, Rebaza A, Sharma L, Cruz CS (2020). Protecting health-care workers from subclinical coronavirus infection. Lancet Respir Med.

[11] Steel RG, Torrie JH (1980). Principles and procedures of statistics, second edition. McCraw-Hill Inc., New York, New York, USA.

[12] Corman VM, Landt O, Kaiser M, Molenkamp R, Meijer A, Chu DK (2020). Detection of 2019 novel coronavirus (2019-nCoV) by real-time RT-PCR. Euro Surveill.

[13] Zhu N, Zhang D, Wang W, Li X, Yang B, Song J (2020). A novel coronavirus from patients with pneumonia in China, 2019. N Engl J Med.

[14] Carlos WG, Dela Cruz CS, Cao B, Pasnick S, Jamil S (2020). Novel Wuhan (2019-nCoV) Coronavirus. Am J Respir Crit Care Med.

[15] Ali S, Noreen S, Farooq I, Bugshan A, Vohra F (2020). Risk assessment of healthcare workers at the frontline against COVID-19. Pak J Med Sci.

[16] Donnelly CA, Malik MR, Elkholy A, Cauchemez S, Van Kerkhove MD (2019). Worldwide reduction in MERS cases and deaths since 2016. Emerg Infect Dis.

[17] Rothe C, Schunk M, Sothmann P, Bretzel G, Froeschl G, Wallrauch C (2020). Transmission of COVID-19 infection from an asymptomatic contact in Germany. N Engl J Med.

[18] Ahmed N, Maqsood A, Abduljabbar T, Vohra F (2020). Tobacco smoking a potential risk factor in transmission of COVID-19 infection. Pak J Med Sci.

[19] Lee CW (2002). Evolution of avian infectious bronchitis virus:genetic drift and recombination. Korean J Vet Serv.

[20] Steinman RM (1991). The dendritic cell system and its role in immunogenicity. Ann Rev Immunol.

[21] Mehta P, McAuley DF, Brown M, Sanchez E, Tattersall RS, Manson JJ (2020). HLH Across Speciality Collaboration. COVID-19:Consider cytokine storm syndromes and immunosuppression. Lancet.

[22] Chen G, Wu D, Guo W, Cao Y, Huang D, Wang H (2020). Clinical and immunological features of severe and moderate coronavirus disease 2019. J Clin Investig.

[23] Wang F, Hou H, Luo Y, Tang G, Wu S, Huang M (2020). The laboratory tests and host immunity of COVID-19 patients with different severity of illness. JCI Insight.

[24] Zhu N, Zhang D, Wang W, Li X, Yang B, Song J (2020). A novel coronavirus from patients with pneumonia in China, 2019. N Engl J Med.

[25] Lu Y, Zhou J, Mo Y, Song S, Wei X, Ding K (2021). Characteristics of chest high resolution computed tomography images of COVID-19:A retrospective study of 46 patients. Pak J Med Sci.

